# General health status of a sample of patients with periodontitis in a Spanish university dental clinic: A case-control study

**DOI:** 10.4317/jced.62102

**Published:** 2024-10-01

**Authors:** Virginia Sánchez, Gema Cidoncha, Miguel de Pedro, Ana Antoranz

**Affiliations:** 1 Department of Clinical Dentistry. Faculty of Biomedical Sciences. European University of Madrid. Spain

## Abstract

**Background:**

During the past years, a bidirectional relationship has been proved between periodontitis and some systemic disorders, such as diabetes mellitus (DM) and cardiovascular diseases (CVDs). The aim of this study was to compare the general health status between patients with and without periodontitis from a Spanish university dental clinic.

**Material and Methods:**

A retrospective observational case-control study was conducted to achieve the research objective. The random sampling was extracted from the patients who attended to the university dental clinic between January 2017 and December 2020.

**Results:**

A total of 2,714 (44.6% males and 55.4% females, 49 [38-60] years old) were included: 1,363 cases (patients with periodontitis; 47.2% males and 52.8% females; 54.6 ± 13.4 years old) and 1,351 controls (patients without periodontitis; 41.9% males and 58.1% females; 44.2 ± 13.4 years old). Patients with periodontitis had lower oral hygiene habits than controls: the 28.9% vs 7.0% (*p*-value <0.001) brushed their teeth once a day, 94.9% vs 69.6% (*p*-value <0.001) did not use interproximal cleaning. 31.0% of periodontal patients were smokers vs 21.2% of the control group (*p*-value <0.001). 51.8% of patients with periodontitis were taking medication vs 31.2% of the controls (*p*-value <0.001). Regarding their general health status, 8.2% periodontitis patients had DM vs 3.9% of the controls (*p*-value <0.001) and 21.3% of the cases suffered from high blood pressure (HBP) vs 10.6% (*p*-value <0.001). In addition, a multivariable regression analysis was performed, where the variables with more strength were age, number of cigarettes and oral hygiene habits.

**Conclusions:**

In the present research, statistically significant differences have observed between patients with and without periodontitis, regarding medication, health problems such as DM, HBP, CVD and cholesterol.

** Key words:**Periodontitis, systemic diseases, health status, university dental clinic.

## Introduction

The workshop conducted in 2017 by the American Academy of Periodontology (AAP) and the European Federation of Periodontology (EFP) in Chicago, titled the “World Workshop on the Classification of Periodontal and Peri-Implant Diseases and Conditions”, introduced a new definition and classification of periodontitis. Periodontitis is defined as a chronic multifactorial inflammatory disease associated with the accumulation of dental plaque (1). This pathology is accompanied by the loss of the teeth-supporting structures, including the alveolar bone and periodontal ligament (2). The disease involves a complex bacterial interaction that triggers a host immune response, but there are also risk factors, such as tobacco use or systemic diseases, that contribute to the progression of periodontitis (3).

Trindade *et al*. (4) conducted a meta-analysis of periodontal epidemiological studies between 2011 and 2020, which was published in 2023. The overall prevalence of periodontitis was estimated at 61.6%.

Patients who suffer from periodontitis often have multiple risk factors, but removing even one of these factors can aid in prevention and treatment and potentially improve their general health (5). Environmental factors, such as tobacco use, can cause alterations in the immune system and promote bacterial growth, while poor oral hygiene can increase bacterial plaque (6,7). Additionally, periodontitis can be related to a patient´s general health; for example, uncontrolled diabetes mellitus (DM) can increase the loss of teeth-supporting structures (8). Conversely, the inflammation promoted by periodontitis can produce an immune response with cardiovascular consequences (9).

The hypothesis of the present study was that the loss of periodontal attachment is linked with systemic disorders. Therefore, the aim of this study was to analyze the general health status of patients with periodontitis and compare it with a control group at European University of Madrid (UEM) Dental Clinic.

## Material and Methods

-Study design

An observational retrospective case–control study was conducted among the patients who attended the European University Dental Clinic in Madrid, from January 2017 to December 2020. All patients signed an informed consent form specifying that all collected data could be used for teaching and scientific purposes.

This study was approved by the UEM Ethics Board (CIPI 20.223) according to the principles of Organic Law 3/2018, 5th of December, of Personal Data Protection and digital rights, and the Royal Decree 1720/2007, Law 41/2002, 14th November, regulating patient autonomy and rights and obligations regarding clinical information and documentation.

-Participants

The participants were classified as cases (patients with periodontitis) and controls (subjects with periodontal health). As guided by the 2017 periodontal classification, a patient must have at least interdental clinical attachment loss (CAL) in two or more contiguous teeth or vestibular CAL ≥3mm with >3mm of probing depth in two or more teeth (2) to be consider periodontal patient. UEM clinical protocols, which are based on this recent classification, were used to define cases as patients with periodontitis and controls as those with periodontal health.

The UEM clinical protocols guidelines are strictly followed by all the university members. When a patient arrives for their first visit, students must take two bitewing radiographs and probe one tooth per sextant, assessing probing depth, bleeding on probing, and horizontal/vertical bone loss. If patients have radiographic bone loss, > 4mm of probing depth and bleeding, they will return for a full periodontal examination. These dental records are double-checked by a professor to verify the proposed treatment plan.

Participants were chosen according to the inclusion/exclusion criteria. Patients had to be over 18 years old and have more than 10 teeth to be included in the study. Participants were excluded if they had an incomplete medical history record, lacked initial diagnosis radiographs, had previous surgical periodontal treatment, or had undergone prior basic periodontal treatment outside UEM dental practice.

-Sample size

A minimum of 1,350 patients per group was established to detect a significant difference between patients with and without periodontitis, with a 95% confidence level and 80% statistical power in a bilateral test. This calculation assumes that the prevalence of DM among patients with periodontitis is 13.1% and 9.6% in the group of patients without periodontitis, and that the worldwide prevalence of periodontitis is 50% (10). A loss rate of around 5% was included.

-Data collection

The data were collected in a decoupled database to maintain confidentiality. The information was handled in accordance with the personal data protection and digital rights guaranteed by Organic Law 3/2018 (5th December).

All information was extracted from the clinical patient records ( Table 1). At University Dental Clinic of UEM, patients are required to fill out a medical history form, which is verified by the students and teachers and saved in the computer system.

-Statistical methods

For the descriptive analysis, absolute (n) and relative (%) frequencies were used to express the qualitative variables. The mean ± standard deviation (SD), or the median and interquartile range (Q1, Q3), were used to express the quantitative variables based on their parametric behavior.

To analyze the differences between sociodemographic and clinical parameters and collected between cases and controls, a Chi-Square test was performed with continuity connections (for qualitative variables) and Student ‘s t-test or Mann-Whitney test (for quantitative variables, depending on whether they had a normal distribution or not).

The effects on cases of variables related to general health, medication intake, and periodontal disease status were assessed using a logistic regression model, and the results were presented as odds ratios (OR) and their corresponding 95% confidence intervals (95%CI). Multivariable regression models adjusted for age, gender, oral hygiene habits, smoking status, and the number of medications (0/<5/>5) were performed for the analysis of diseases.

Data analysis was carried out with SPSS® version 29.0 software (IBM Corp., USA). The level of statistical significance was set at *p* < 0.05.

## Results

**Figure 1:**
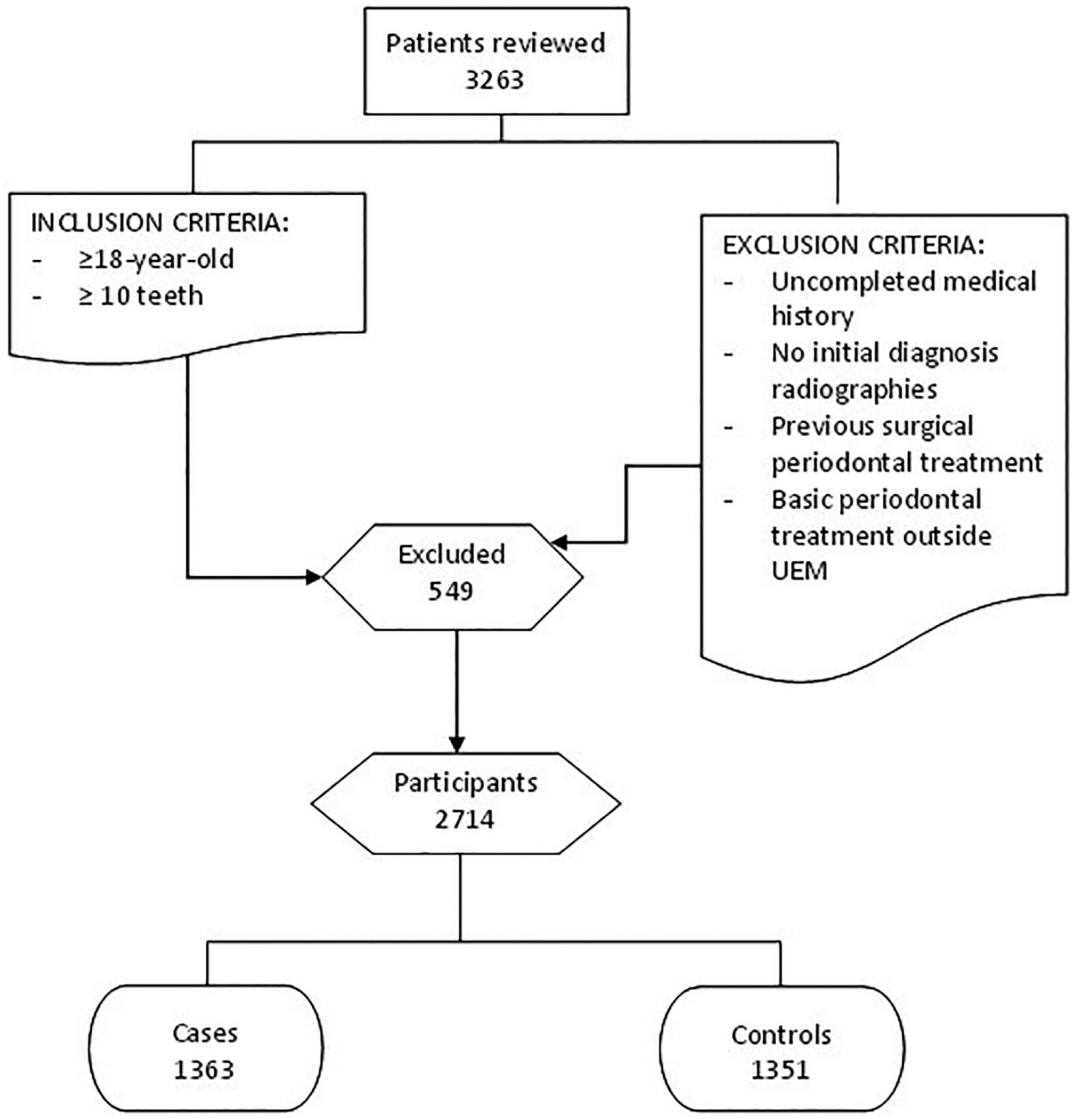
Selection patient´s flowchart.

As shown in Figure 1, a total of 3,263 patients were reviewed. Following the inclusion / exclusion criteria, 549 participants were excluded. The sample consisted of 2,714 participants, with 1,363 cases and 1,351 controls.

The study population comprised 55.4% females and 44.6% males, with a median age of 49 (38-60) years old. Regarding smoking habits, 26.2% of the group were smokers, with a median of 10 cigarettes consumed by those who smoked. Regarding oral hygiene, 4.2% of the study´s patients did not brush their teeth, while 23.7% brushed three times a day; 82.3% of the sample did not use interproximal cleaning. In terms of medication, the 41.5% were on drug treatment, with 36.1% of the patients taking <5 Tablets a day and the 5.4% were consuming ≥5 Tablets.

-Cases and controls´ sociodemographic characteristics and habits.

A significant difference was observed in gender, with a higher proportion of men in cases compared to controls. There was also a significant age difference, with cases being on average 10 years older than controls. Regarding oral hygiene, 12.7% of patients with periodontitis brushed their teeth three times a day and only 5.1% used dental floss, whereas 34.9% of controls brushed three times a day and 30.4% used interproximal cleaning. Among periodontal patients, 31.0% were smokers, with a median consumption of 15 cigarettes per day. All the patients’ characteristics and habits compared between cases and controls were significantly (*p* < 0.05) more frequently reported by periodontal patients ( Table 2).

The prevalence of systemic diseases in cases and controls.

Self-reported systemic medical conditions examined and compared between cases and controls are presented in  Table 3. Six medical conditions were considered. Diabetes mellitus (DM), high blood pressure (HBP), cerebrovascular disease, peripheral arterial disease, and cholesterol were significantly more frequently reported by cases. The prevalence of coronary heart disease did not significantly differ between cases and controls.

The results of the multivariable regression analysis for the association between systemic diseases and periodontitis, adjusted for age, sex, number of medications, smoking habits, and oral hygiene habits, are shown in  Table 4. A higher proportion of individuals reported high cholesterol levels and medication use in cases compared to controls. In contrast, DM and cardiovascular diseases (HBP, coronary heart disease, cerebrovascular disease, peripheral arterial disease) were not significantly associated with periodontitis.

## Discussion

-Influence of patients’ general characteristics 

According to the present results, age could be considered a risk factor, as there was a higher percentage of patients over 55 years old in the periodontitis group compared to the control group. The study findings are consistent with research conducted by Eke *et al*. (11) in the United States, where data from 1,511 older adult patients were collected to determine the prevalence of periodontitis in this age group. They used a regression model to reach their results, demonstrating that periodontitis is prevalent among older adults in the USA. The exact reasons are not entirely clear; authors suggest it may be due to the accumulation of bacterial plaque leading to bone loss (12,13), while other publications suggest it could be attributed to the aggregation of various risk factors, such as smoking status and overall health (2,14).

Gender is considered a risk factor, although the reason for the difference is not entirely clear. According to the cross-sectional study conducted by Ragghianti *et al*. (15) in Brazil, which involved 380 patients to assess the influence of age, sex, plaque, and smoking on periodontal disease, an association was observed between these factors and the increase in periodontal destruction. The possible reason for this link could be a combination of behavioral patterns, genetic factors, and hormonal influences contributing to males having a higher risk of suffering from periodontitis compared to females (16,17).

-Patients’ environmental factors and their association with periodontitis

There are several modifying factors, such as oral hygiene, tobacco use, and pharmacological agents, which could have a negative influence on the periodontium. Regarding patients’ oral hygiene habits, the present study showed that both cases and controls brushed their teeth an average of twice a day, and a large percentage of periodontal patients (94.9%) did not use interproximal brushes or dental floss. This outcome is consistent with other studies where the presence of bacterial plaque, influenced by oral hygiene habits, is considered a risk factor for periodontitis (7,18). Bertelsen *et al*. (19) conducted a study in Norway on self-reported oral hygiene habits similar to those in the present study, examining the microbiome of gingival fluid composition in 484 patients and their community periodontal index score. One of the most interesting aspects of Bertelsen’s study is their exploration of bacterial community composition associated with dental hygiene habits and periodontal diagnosis.

As mentioned earlier, another significant risk factor for periodontitis is smoking status (3). In an epidemiological study by Carasol *et al*. (20) assessing the prevalence of periodontitis among a Spanish population of 5,130 workers based on periodontal examination and sociodemographic factors, higher prevalence was observed among smokers. The link between periodontitis and tobacco consumption in both Carasol’s and the current study may be due to the heat causing periodontal damage leading to increased attachment loss, and nicotine altering immune responses and enhancing anaerobic bacterial growth through vasoconstriction (21).

In addition to the aforementioned risk factors, drug-induced gingival enlargement has been well-documented in recent years. Certain medications such as antiepileptics, phenytoins, cyclosporins, or calcium channel blockers are associated with gingival enlargement (7,22). Wang *et al*. (23), in 2020, analyzed 33 drugs comparing patients with and without periodontitis, finding associations between medications used for cardiovascular disease (CVD) or DM similar to those in the present study. This could be attributed to these medications being used for conditions related to periodontal attachment loss, which will be further explained below.

-Relationship between periodontitis and patients’ general health status

Patients´ general health status can have a negative impact on the periodontium presence or, at least, can influence on periodontitis ‘course. In the present study, 4 of the most common systemic diseases have been perused.

According to the 2020 National Health Statistics of Spain, one of the most prevalent chronic diseases is high cholesterol, affecting 15.0% of the population (24). Penumarthy *et al*. (25) conducted a clinical study involving 90 subjects (30 periodontally healthy, 30 with gingivitis, and 30 with periodontitis), performing both periodontal exams and blood tests to understand the influence of periodontitis on lipid metabolism. Although our study did not include blood tests and relied on self-reported medical histories, we observed that patients with high cholesterol were at higher risk of periodontitis. This association could be attributed to the modification of proinflammatory cytokine production, which plays a role in the immune response (26).

There is strong evidence of an association between CVD and periodontitis (27). Similar to the present study, Sumayin *et al*. (28) conducted a cross-sectional study in the United States based on the new periodontal classification, aiming to examine the relationship between periodontitis and CVD, demonstrating a significant association. This correlation may be due to oral microbiota entering the bloodstream and eliciting an inflammatory response (29,30), or potentially shared genetic factors influencing irregular inflammatory responses in both conditions (27). Regarding the relationship between HBP and periodontitis, the pathogenesis is similar to that of other CVDs. Könnecke *et al*. (31), performed a large population-based health survey with periodontal examinations and blood pressure measurements, finding an association between periodontitis and HBP independent of age, sex, DM, or smoking. This relationship may stem from chronic inflammation around tooth-supporting tissues triggering an immune response that in turn affects blood pressure (9). Periodontitis, HBP, and other CVDs could share immune response genes and other risk factors such as tobacco use, gender, or age (32).

The final pathology analyzed was DM, a common condition in Spain affecting approximately 8% of the population according to the 2020 National Health Statistics (24). The results of our study align with other studies suggesting DM as a risk factor for periodontitis, and vice versa (33,34). Tooi *et al*. (33) in a cross-sectional study involving medical records and full-mouth probing depth measurements of 150 participants, found an association between probing depth and patients with mild DM. The biological link between these two conditions is well-established; hyperglycemia leads to the formation of advanced glycation end products (AGEs) and chronic inflammation causing tissue damage and preventing periodontal tissue healing. Poorly controlled glycemia also induces microvascular changes in the gingiva, exacerbating periodontal inflammation (35). It is crucial for patients, endocrinologists, and dentists to recognize this bidirectional relationship to prevent periodontitis and improve the health of diabetic patients.

Multivariable regression analysis was conducted to examine the relationship between systemic diseases, medications, and periodontitis within the same context. The findings indicated that older age, smoking more than 10 cigarettes per day, or inadequate tooth brushing had a stronger association with periodontitis than suffering from a systemic disease. However, in the study population, individuals with health disorders were more likely to have periodontitis, potentially due to older patients experiencing more general health issues, taking medications, and the cumulative effects of poor oral hygiene and tobacco use over the years.

A strength of this investigation was its large sample size of 2,714 patients. This study explored potential associations between systemic diseases, general health status, age, gender, smoking status, and oral hygiene habits with periodontitis. Currently, there is limited comprehensive data available in the literature regarding the collective impact of all these variables on periodontitis. The evidence for potential relationships was derived from a substantial database of a university dental clinic.

The primary limitation of this study is that all data collected on medications, systemic diseases, oral hygiene habits, and smoking status were self-reported. This could lead to under-diagnosis, as some patients may not be aware of all their conditions or medications. Furthermore, there is potential for patients to misreport tobacco consumption or oral hygiene habits.

In conclusion, a statistically significant relationship was observed between general health status and periodontitis. The findings support the notion that the progression of periodontal disease can be influenced by risk factors such as age, smoking, oral hygiene habits, medication use, and systemic diseases. However, further high-quality studies are needed to definitively establish these factors as periodontal risk factors. Future research should focus on examining the temporal relationship between the presence of these risk factors and the development of periodontitis.

## Figures and Tables

**Table 1:** Variables of the study. Abbreviations: DM- Diabetes mellitus; HBP- high blood pressure.

VARIABLE	DEFINITION	MEASUREMENT UNITS
Periodontitis	Loss of teeth supporting structure	Yes/No
Age	Age of the patient at the time of diagnosis	18-24
		25-34
		35-44
		45-54
		≥55
Gender	Patient's biological sex	Male/female
Smoking	Patient´s tobacco use	Yes/No/Former smoker
Number of cigarettes	Number of cigarettes consumed per day	1-10
		11-20
		21-30
		+30
Toothbrushing	Number of times the patient brushes daily	0/1/2/3
Interproximal cleaning	Use of interproximal cleaning methods/aids	Yes/No
Medications	Patient is on prescribed medication	Yes/No
Number of medicines	Number of medicines the patient takes per day	0
		<5
		≥5
DM	Patient suffers from diabetes mellitus	Yes/No
HBP	Patient suffers from hypertension	Yes/No
Coronary Heart Disease	Patient suffers from coronary heart disease	Yes/No
Cerebrovascular disease	Patient suffers from cerebrovascular disease	Yes/No
Peripheral arterial disease	Patient suffers from peripheral arterial disease	Yes/No
Cholesterol	Patient suffers from cholesterol	Yes/No

Abbreviations: DM- Diabetes mellitus; HBP- high blood pressure.

**Table 2:** Characteristics and patients’ habits compared between cases and controls.

	Patients with periodontitis	Control patients	p-value
	(n= 1363)	(n= 1351)	
Aged			
Mean	54.60	44.20	< 0.001^a^
SD	±13.40	±16.10	
Gender			
Male, n= 1210	644 (47.20%)	566 (41.90%)	0.005^b^
Female, n=1504	719 (52.80%)	785 (58.10%)	
Smoking			
Yes, n=710	423 (31.00%)	289 (21.20%)	< 0.001^b^
No, n= 1760	778 (57.10%)	982 (72.70%)	
Former- smoker, n= 224	162 (11.90%)	82 (6.10%)	
Number of cigarettes			
Mean	6.40	2.70	<0.001^a^
SD	±9.10	±5.70	
Interproximal cleaning			
No, n=2233	1293 (94.90%)	940 (69.60%)	<0,001^b^
Yes, n= 482	70 (5.10%)	411 (30.40%)	
Tooth brushing			
Zero, n= 113	110 (8.10%)	3 (0.30%)	<0,001^b^
One, n=489	394 (28.90%)	95 (7.00%)	
Two, n=1467	686 (50.30%)	781 (57.80%)	
Three, n=645	173 (12.70%)	472 (34.90%)	

Abbreviations: SD-standard deviation, n- sample size, %- percentage, a - T – student test, b - Pearson´s Chi-Square

**Table 3:** Patients’ medical status and medication. Comparison between cases and controls.

	Patients with periodontitis	Control patients	p-value
	(n= 1363)	(n= 1351)	
DM, n (%)	112 (8.20)	53 (3.90)	<0.001^b^
HBP, n (%)	291 (21.30)	143 (10.60)	<0.001^b^
Coronary heart disease, n (%)	14 (1.00)	6 (0.40)	0,076^b^
Cerebrovascular disease, n (%)	18 (1.30)	6 (0.40)	0.015^b^
Peripheral arterial disease, n (%)	14 (1.00)	4 (0.30)	0.019^b^
Cholesterol, n (%)	181 (13.30)	58 (4.30)	<0.001^b^
Medication			
No, n= 1587	657 (48.20%)	930 (68.80%)	< 0.001^b^
Yes, n= 1127	706 (51.80%)	421 (31.20%)	
Number of medications			
Mean	1.40	0.70	<0.001^a^
SD	±2.30	±1.60	

Abbreviations: n- sample size, %- percentage, a - T – student test, b - Pearson´s Chi-Square, SD-standard deviation.

**Table 4:** Multivariable regression analysis for periodontitis,including systemic medical conditions, number of medications, oral hygiene habits, smoking habits, age and sex.

	Adjusted OR (95% CI)	p-value
Age		
25-34	2.453 (1.331 - 4.520)	0.004
35-44	7.973 (4.507 - 14.104)	< 0.001
45-54	10.753 (6.133 - 18.851)	< 0.001
≥55	14.634 (8.305 - 25.784)	< 0.001
Gender		
Female	1.134 (0.933 - 1.378)	0.206
Smoking		
Former smoker	1.883 (1.355 - 2.615)	<0.001
< 10 cigarettes / day	1.004 (0.723 - 1.395)	0.979
≥10 cigarettes / day	2.469 (1.903 - 3.203)	<0.001
Tooth brushing		
2	1.751 (1,360 - 2.162)	<0.001
1	6.983 (5.043 – 9.669)	<0.001
0	66.226 (19.962 – 219.718)	<0.001
Interproximal cleaning		
Yes	0.186 (0.139 – 0.251)	<0.001
Number of medications		
<5	1.387 (1.117 – 1.721)	0.003
≥5	1.503 (0.950 – 2.378)	0.081
DM		
Yes	0.895 (0.581 – 1.379)	0.651
CVD		
Yes	0.864 (0.635 – 1.174)	0.350
Cholesterol		
Yes	1.613 (1.112 – 2.338)	0.012

Abbreviations: CI 95%- confidence interval, OR- odds ratio.

## Data Availability

The datasets used and/or analyzed during the current study are available from the corresponding author.
